# Theodor Hellbrügge: 85 years of age – Ad multos transannos, sanos, fortunatos et beatos

**DOI:** 10.1186/1740-3391-3-2

**Published:** 2005-03-05

**Authors:** Franz Halberg, Germaine Cornélissen, George Katinas, Othild Schwartzkopff, Dana Johnson

**Affiliations:** 1 Halberg Chronobiology Center, University of Minnesota, Minneapolis, MN 55455, USA

## Abstract

We honor Theo Hellbrügge's acclaimed endeavors in the rehabilitation, or rather the prehabilitation of handicapped children. So far, he has focused on obvious handicaps, and we trust that he will include concern for everybody's silent handicaps in the future by screening for abnormal variability inside the physiological range. Therein, we introduce cis- and trans-years, components of transdisciplinary spectra that are novel for biology and also in part for physics. These components have periods, respectively, shorter and longer than the calendar year, with a counterpart in magnetoperiodism. Transyears characterize indices of geomagnetic activity and the solar wind's speed and proton density. They are detected, alone or together with circannuals, in physiology as well as in pathology, as illustrated for sudden cardiac death and myocardial infarction, a finding calling for similar studies in sudden infant death syndrome (SIDS). As transyears can beat with circannuals, and depend on local factors, their systematic mapping in space and time by transdisciplinary chronomics may serve a better understanding of their putative influence upon the circadian system. Longitudinal monitoring of blood pressure and heart rate detects chronome alterations underlying cardiovascular disease risk, such as that of myocardial infarction and sudden cardiac death. The challenge is to intervene in a timely fashion, preferably at birth, an opportunity for pediatricians in Theo Hellbrügge's footsteps.

## Laudatio

The discovery in biology of far-transyears, 15–20 months in length [1-3], is in keeping with oscillations of the same longer-than calendar-yearly period in the speed and proton density of the solar wind [4,5]. Hence, this wish for healthy, lucky and blessed transyears rather than years. Let us speculate that we are genetically programmed for a certain number of transyears (or years) and that an attempt to synchronize transyears rather than years, also pure speculation, could automatically prolong the remaining lifespan by one or two-thirds in the case of far-transyears or by some weeks in the case of a near-transyear. What is not speculation is that transyears are a transdisciplinary fact of life and that they can beat with a spectral component with a period of the length of the calendar year [1-3], and, what seems critical for this journal, each about-yearly component can influence the circadian system.

**Figure 1:**
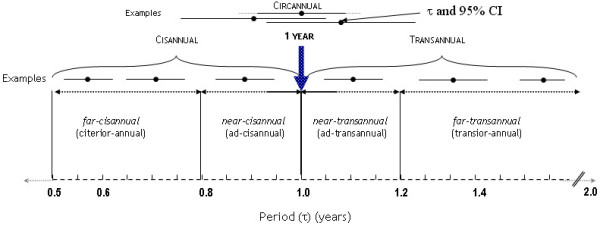
**Tentative scheme for classification of cis- and trans-yearly periods, based on length and 95% confidence interval (CI), without implication as to mechanisms**. Period (τ, dot), with its 95% CI (length of horizontal line), indicated for near and far trans- and cis-yearly components in transdisciplinary, including physical-environmental and biologic spectra, the latter at all levels of organization, from single prokaryote to ecosystems. Circannual (about calendar-yearly) components under usual conditions are defined as components with a τ, the 95% CI of which overlaps the precise yearly τ; trans- and cisannuals are components with a 95% CI of τ not overlapping the precise yearly τ, longer (trans) or shorter (cis) than 1 year, respectively, with distant limits indicated on the scheme. They are subdivided further into near- and far- cis- or transyears, if the 95% CIs are within the limits also shown on this graph.

Figure 1 presents a tentative scheme for classification of trans-yearly spectral components. The suggestions are tentative; they imply that the cis- and trans-annuals, as defined here, have an amplitude (A) different from zero, established by the non-overlap of zero by the 95% confidence interval (CI) of A, and that the component is anticipated, i.e., confirmed by analyses of an independent separate prior series. In addition to these considerations of statistical significance and prior documentation, there is a most important added consideration of reciprocal mutually supporting cyclicities found in and around us. These are much more numerous in the case of the spectral region around the year than in that of the day. Moreover, about-yearly cycles, notably the non-photic magnetoperiodisms, usually are mere influencers of the biological year, rather than necessarily long-term synchronizers, being often transients themselves, by contrast to cycles with a period corresponding in length to the day. In the case of the year, the far-transyears centering around 1.3 years and around 1.6 years are all different and transient, and, this is new, their influence is also dependent upon local factors. The far-transyears were discovered by physicists in the solar wind with prior hints from geomagnetics and auroral counts [4,5] while the near-transyears in the solar wind, in the antipodal geomagnetic index as well as in biology, were found and validated by us. Because of the wobbliness of the period and the circumstance that the external cycles may not lock-in the biological ones, variability is much greater in the about-yearly spectral region than in the circadian domain. In the case of the about-yearly vs. that of the about-daily variation, about-yearly asynchronization must be considered rather than desynchronization, as in the case of circadians.

For discussion by transdisciplinary nomenclature committees, terms in English are emphasized. With advice by Prof. Robert Sonkowsky, proposed Latin equivalents are added for vanishing classicists. Essentially, "ad-transannual" means "a little longer than a year"; "ad-cisannual" means "a little shorter than a year"; "transior-annual" means "much longer than a year"; and "citerior-annual" means "much shorter than a year". Some specific limits that seem reasonable in the light of available physical and biological evidence are given in the scheme. The single syllable 'ad' is preferred to the 2-syllable 'prope', 'juxta', 'propter', 'minus' (paired with 'plus') or the 3- or 4-syllable 'proprior', 'proximus', 'vicinus', or propinquus'. While to a purist among grammarians the coinages adtransannual and adcisannual may seem preposterous (a word constituting itself an illustration of cumulative prefixes) precisely because of the piling on of prefixes, there are also other precedents in Late Latin such as exinventio ("discovery") and perappositus ("very suitable/apposite"). Normal assimilation of 'd' to 't' and 'c', respectively, may then result in the spellings and pronunciations "attransannual" [at-trans-annual] and "accisannual" [ak-sis-annual] acceptable as English pronunciation, notably by speakers with native romance languages, who may face difficulty with the near and far as added prefixes.

Difficulties may stem from the fact that analyses usually provide estimates in frequency (not period) terms, and from the criterion of 95% CIs that may not be available. We need to allow for situations when, because of too-wide (or unavailable) CIs, we can diagnose only a candidate trans- or cis-annual component, when 95% CIs of τ overlap the limit distant from the year. By the same token, we may not be able to specify near or far, e.g., because of the brevity of the series. In other words, we cannot say whether we have a near- or a far- trans- or near- or far- cis-year, when there is an overlap by 95% CIs with the corresponding finer limits, shown on the scheme (Figure 1).

For the case of "circannual", we again go by 95% CIs rather than by the point estimate. In the circannual case, the 95% CI overlaps the 1-year estimate under usual conditions, bearing in mind that under unusual, e.g., constant conditions, circannuals are also amenable to free-running, in which case the 95% CI may no longer cover 1 year but will have to be tested further for non-overlap with the pertinent environmental cycle in the case of a biologic cycle and vice versa for non-overlap of a natural environmental cycle with an anthropogenic cycle. In the trans- or cis-annual case, the 95% CI does not cover the 1-year period under usual conditions, i.e., cis- or trans-annuals can be asynchronized rather than desynchronized. Strictly speaking, circannual cannot be an overall term, but almost certainly, whatever committees may decide, it will be (mis-)used as such. "Far-" and "near-", "cis-" and "trans-" and "citerior-" and "transior-" annual are hyphenated here only to indicate their derivation and need not be written with hyphens. We propose using circannual, transannual or cisannual and their refinements, only operationally as a function of periods and their 95% CIs. Matters of synchronization, desynchronization or asynchronization may then possibly emerge from the context of a given situation and from further testing.

Trans- and cis-years lead to a novel chrono-helio-geobiology, awaiting application of the tools of transdisciplinary chronomics. It has been a challenge to look at circadians for the past half-century, but knowledge concerning them will not be completely useful before we answer another set of questions based on the evidence in Table 1.

**Table 1:** Geomagnetic/Geographic Differences among Cycles with Periods in the Range of 0.8 – 2.0 years Characterizing the Incidence of Sudden Cardiac Death and Myocardial Infarction

Sudden Cardiac Death (SCD)^1^*									
Site	Span	T, Δt, N	SC (N)	Period (y)	(95%CI)	Amplitude	(95%CI)	A(% MESOR)	P-value^2^
**Transyear (TY) or Candidate Transyear (cTY) Detected**									
Minnesota	1999–2003	5 y, 1 d, 1826	343	1.392 (TY)	(1.173, 1.611)	0.042	(0.00, 0.09)	22.0	0.014
Arkansas	1999–2003	5 y, 1 d, 1826	273	1.095	(0.939, 1.251)	0.032	(0.00, 0.07)	21.1	0.040
				1.686 (cTY)	(1.293, 2.071)	0.031	(0.00, 0.07)	20.7	0.044
Czech Rep.	1999–2003	5 y, 1 d, 1826	1006	0.974	(0.856, 1.091)	0.078	(0.00, 0.16)	14.2	0.007
				1.759 (cTY)	(1.408, 2.110)	0.077	(0.00, 0.15)	13.9	0.010
	1994–2003	10 y, 1 d, 3652	1792	1.726 (TY)	(1.605, 1.848)	0.074	(0.02, 0.13)	15.1	<0.001
				1.000	(0.944, 1.056)	0.052	(0.00, 0.10)	10.6	0.010
									
**Candidate Transyear Not Detected**									
North Carolina	1999–2003	5 y, 1 d, 1826	752	0.929	(0.834, 1.023)	0.069	(0.00, 0.14)	16.9	0.007
Tbilisi, Georgia	Nov'99–2003	4.1 y, 1 d, 1505	130	0.988	(0.862, 1.114)	0.035	(0.00, 0.07)	40.7	0.007
Hong Kong	2001–2003	3 y, 1 m, 36	52	0.843	(0.651, 1.036)	0.022	(NS)	44.9	0.077

**Myocardial Infarction (MI)**									
Site	Span	T, Δt, N	MI (N)	Period (y)	(95%CI)	Amplitude	(95%CI)	A(% MESOR)	P-value^2^

**Coexisting Year (Circannual) and Transyear (TY)**									
Czech Rep.	1999–2003	5 y, 1 d, 1826	52598	1.014	(0.989, 1.038)	2.85	(2.22, 3.48)	9.88	<0.001
				1.354 (TY)	(1.252, 1.456)	1.35	(0.69, 2.02)	4.68	<0.001
	1994–2003	10 y, 1 d, 3652	115520	0.998	(0.988, 1.009)	3.03	(2.47, 3.60)	9.58	<0.001
				1.453 (TY)	(1.417, 1.489)	1.91	(1.34, 2.49)	6.04	<0.001
				1.15 (TY)	(1.116, 1.184)	1.23	(0.64, 1.82)	3.88	<0.001

* With focus on transyears with periods longer than 1.0 year.

^1^International Classification of Disease (ICD10) Code I46.1, excluding MI and sudden death of unknown or unspecified cause (except before 1999). T: Length of data series (y = years); Δt: sampling interval (d = day, m = month); N: number of data (including 0s). Period and 95% confidence interval (CI) estimated by nonlinear least squares. In longer (10-y) series, a neartransyear (cycle with a period between 1.0 and 1.2 y) is detected for MIs in addition to a fartransyear. Brevity of series and lack of ordering statistical significance qualify results from Hong Kong. Note that transyears are found in 3 of 6 locations (P < 0.05 by linear least squares) with a relative amplitude >12 (% of MESOR).

^2^From linear least squares analysis, not corrected for multiple testing. Amplitude expressed in N/day.[62]

Table 1 demonstrates in the incidence of myocardial infarction (MI) in the Czech Republic and, for sudden cardiac death (SCD), in the strict sense, excluding MI, both a calendar year and a candidate transyear component in Arkansas as well as in the Czech Republic yet only a transyear, no calendar year for SCD in Minnesota. Signatures and thus perhaps a putative influence of magnetic cycles on human SCD constitute a new feature of SCD pathology, which gains in prominence when death from MI and from other unknown or unspecified causes is ruled out, as it is likely to be when ICD10 code I46.1 is used, as is the case in Table 1.

Of interest are great geographic/geomagnetic differences insofar as no transyears, only calendar-yearly components, were detected in 3 locations, while in 3 other locations, transyears were present, in two of these, with a coexisting calendar-yearly component, with nearly equal prominence, while in Minnesota, only a transyear was thus far detected. A clarification of the roles played by local as well as global influences could also be based on transyear vs. calendar-yearly amplitude ratios when both components are present, which, however, is not the case in 4 of 6 locations. There is the challenge of developing eventual countermeasures.

But first, we seek a clue as to why, for SCD in Minnesota, the prominence of the transyear exceeds by far any seasonal, thus far undetected influence of the harsh environmental temperature change in its mid-continental climate in the summary of 5 consecutive years, and why, in Arkansas and the Czech Republic, the transyear's prominence is about the same as that of the seasons, and why it seems to be absent in 3 other locations and furthermore why in MI the prominence (gauged by the amplitude) of the calendar year is so far greater than that of the transyear (by contrast to the case of SCD). Systematically collected data from different areas of the world will open a new chapter in transdisciplinary science, with particular pertinence at the extremes of extrauterine life, in natality as well as in mortality.

Optimization of the about-yearly spectral region may also be considered, along with Hufeland's consideration of the daily routine in studies aimed at prolonging high-quality life [6]. Notably in the baby, but also in the elderly, the far-transyear's amplitude can exceed that of a spectral component with the length of a calendar year, and hence transyears are especially important to pediatricians and geriatricians alike and, perhaps, for scholars in the field of circadian rhythms.

**Figure 2:**
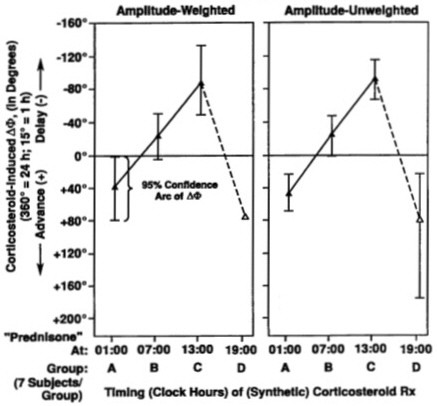
**Importance of timing treatment: Phase shift (ΔΦ) of peak expiratory flow (PEF) rhythm as a function of timing of prolonged corticosteroid therapy in children with severe asthma**. Drastic differences in direction and extent of drug-induced shift of a circadian acrophase as a function of medication timing. The reference phase (0°) is the phase of PEF of a group of untreated children with asthma in remission. Vertical 95% confidence intervals indicate detection of statistically significant circadian rhythm (by cosinor) [15].

**Figure 3:**
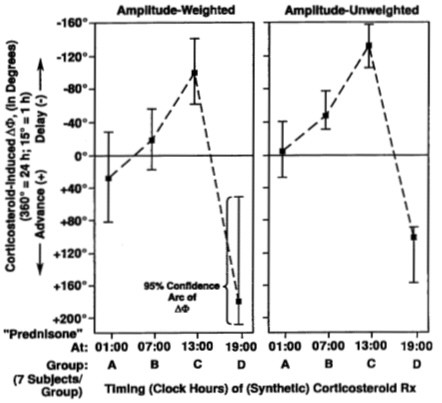
**Importance of timing treatment: Phase shift (ΔΦ) of circadian rhythm in urinary potassium excretion as a function of timing of prolonged corticosteroid therapy in children with severe asthma**. Drastic differences in direction and extent of drug-induced shift of a circadian acrophase as a function of medication timing. The reference phase (0°) is the phase of urinary potassium excretion of a group of children with moderate asthma not treated by corticosteroid. Vertical 95% confidence intervals indicate detection of statistically significant circadian rhythm (by cosinor) [15].

**Figure 4:**
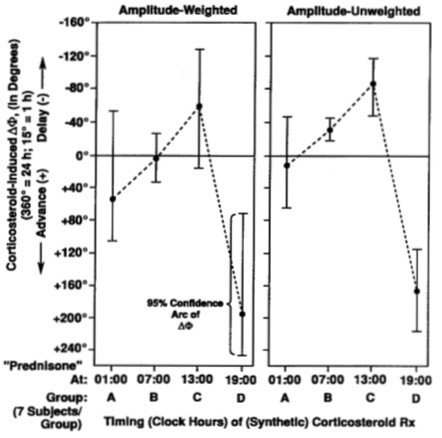
**Importance of timing treatment: Phase shift (ΔΦ) of circadian rhythm in urinary chloride excretion as a function of timing of prolonged corticosteroid therapy in children with severe asthma**. Drastic differences in direction and extent of drug-induced shift of a circadian acrophase as a function of medication timing. The reference phase (0°) is the phase of urinary chloride excretion of a group of children with moderate asthma not treated by corticosteroid. Vertical 95% confidence intervals indicate detection of statistically significant circadian rhythm (by cosinor) [15].

Beyond 85 years of age, Theodor Hellbrügge, chronopediatrician *par excellence *and professor emeritus of social pediatrics at the University of Munich, continues actively as a mentor of the specialty he founded [7-9]. Our earlier laudatios [7,10-14] include a symposium dedicated to Theo [14], which competes with his 2 honorary professorships, 17 honorary doctorates, and many more institutes built for handicapped children after his model center in Munich. Theo started as a solid contributor of chronobiological data, he continued in the field via a school of medical students who wrote their doctoral theses and participated broadly in this field, most of them in Minnesota [15-58], many of them concerned with prehabilitation in terms of vascular disease prevention [24-34,38-47,49,52,53,55]. Methodological papers were critical [15-19] to a time-microscopic inferential statistical assessment of both drug-induced phase shifts and circadian phase-response maps, given in each case with the uncertainties involved (Figures 2, 3, and 4) [15].

**Figure 5:**
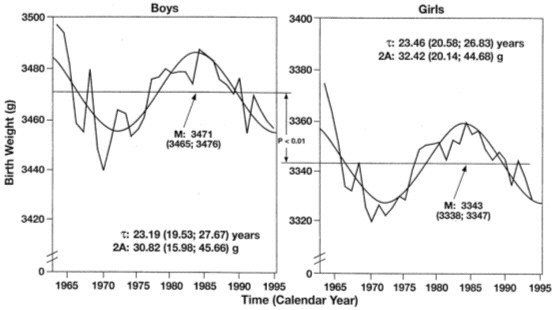
**"Secular" trends in birth statistics from Minnesota uncovered as putative testable cosmic signatures**. Shown are the residuals from second-order polynomial fit. Period (τ), double amplitude (2A) and MESOR (chronome-adjusted mean value) assessed by nonlinear least squares, listed with 95% confidence limits. Birth weight in Minnesota undergoes changes that could be signatures during evolution and/or contemporaneously of the cycle in sunspot bipolarity (N of babies: 2,136,745 = 1,097,283 boys and 1,039,462 girls).

**Figure 6:**
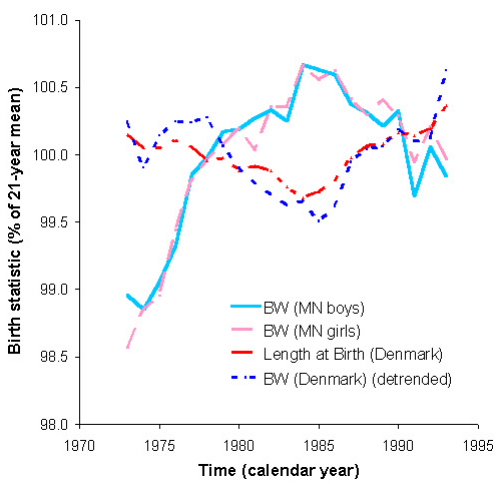
**Geographic/geomagnetic differences? Near-antiphase of circadidecadal changes in neonatal body weight (BW) in Minnesota (MN) (N = 2,136,745 babies) or neonatal body weight and length in Denmark (N = 1,166,206 babies)**. Putative signatures of the Hale bipolarity cycle of sunspots are in antiphase. Did K.F. Gauss anticipate geographic/geomagnetic differences due to the little but close magnet Earth itself, reversing the phase of a putative effect upon the period of the large yet far magnet Sun, when Gauss, like A. von Humboldt, each started mapping geomagnetics at different latitudes?

**Figure 7:**
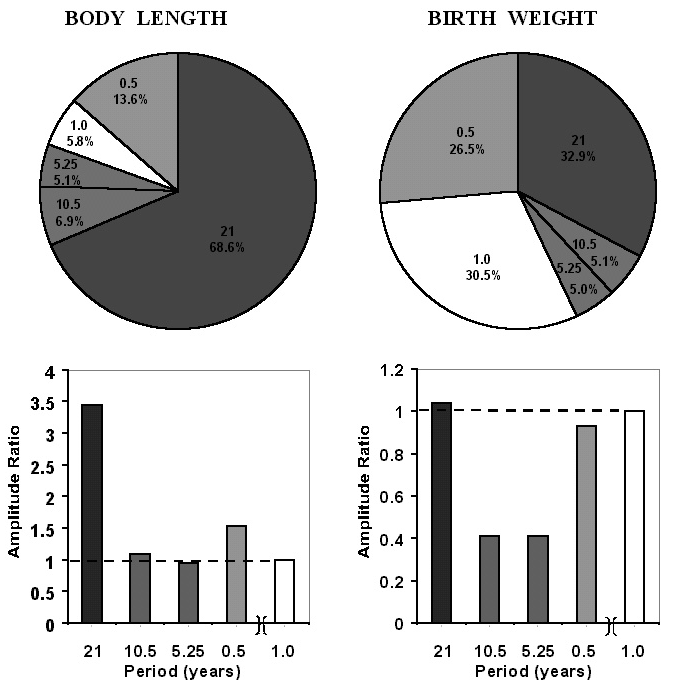
**What we do not see can be more important than the visible: Relative contribution of mainly non-photic (shaded) versus mainly photic (white) spectral components in human neonates**. The extent of change (double amplitude) of the non-photic, probably circadidecadal Hale cyclicity, a signature of sunspot bipolarity, can exceed that of the usually solely considered yearly component to the population pattern of human neonatal body length. Amplitude ratios were assessed by the variance of each selected component given as percentage of their sum (top) and as amplitude ratios (bottom). Linearly determined is the relative prominence of biological counterparts of about 21-year (Hale) and about 10.5-year (Schwabe) solar activity cycles, with a 5.25-year harmonic assessed to account for any non-sinusoidality; 0.5-year component is counterpart of geomagnetic disturbance cycle. Meta-analysis of Danish National Birth Registry for all children (N = 1,166,206) born from 1973 to 1994 (*The Lancet *1998, 352 (26): 1990).

Theo himself turned in the interim to the care of children with obvious disabilities. He continues with concerns about them to detect early alterations for timely remedies, a preventive task *par excellence*, which could benefit from chronomics, the resolution of time-structural (chronome) alterations in the physiological range. Accordingly, chronobiologists honored Theo at a meeting on "Time structures – chronomes – in child development", leading to a proceedings volume of 256 pages [14]. On the basic side, this conference documented that the human newborn may recapitulate the development of life on earth by a chronome different from that of an adult. The amplitude of about 7-day vs. about-24-hour variation in the human circulation has been shown in gliding spectra in this journal earlier [59]. The amplitudes of spectral components' longer-than-yearly periods can be more prominent than about-yearly changes [14]. About 21-yearly cyclicities (Figure 5) pose interesting problems of geographical differences [14]. These about 21-year cycles correspond in period length to the sunspots' bipolarity cycle [60], but are nearly in antiphase in Minnesota vs. Denmark (Figure 6), raising the question of how different aspects of the earth's surface may bring about antiphasic responses to putative non-photic solar effects, with contributions that are hardly negligible (Figure 7). Possible geomagnetic or other environmental effects on the period and thus indirectly on the phase are implied in Table 1 with respect to sudden cardiac death in a strict sense, excluding death from MI [62]. In conjunction with chaos and trends – in chronomes – these complex cycles provide insight into many developmental biological processes and behavioral patterns in infancy and childhood [14] and also at the other end of life [62] (Table 1).

In his own recent words [63], Theo also "had an interest in the work in Prague of pediatricians and psychologists like Matajcek, Dolanski and Donovski, who were interested in systematically analyzing a neonatal deprivation syndrome. From their lessons, [Theo] formulated the concept of developmental rehabilitation in Munich, with new programs for early diagnosis, early therapy and early incorporation into society." In seeking a niche for his endeavor, he called his program "rehabilitation" rather than "prehabilitation" [64,65]. Thus, for his endeavors, he was able to tap into a source of funds already officially earmarked for rehabilitation.

To continue in his words [63], in practice, Theo "used the plasticity of the central nervous system in early childhood to develop a targeted treatment of children who have innate or early-acquired disturbances or actual damage in order to save them from the fate of a lifelong handicap. In so doing, he is proud that he helped completely deaf children, via their mothers, to learn normal speech when they were offered speech treatment in the first weeks and months of life. This concept was extended worldwide and led to the publication of books for parents on 'The First 365 Days of a Child's Life' [8]." Theo believes that "this is the most important discovery of the newest pediatric research, in which Czech and Slovak researchers like Janos Papousek participated and discovered that the newborn is already a very competent 'learning system'." Indeed, the evaluation of hearing loss in infants and young children requires early identification and assessment of hearing impairment, an endeavor of critical importance to cite John Jacobson and Kara Jacobson [66]: "New technology and techniques have helped make the process more efficient and accurate for pediatricians."

By 1960 at Cold Spring Harbor [67] and again thereafter at the New York Academy of Science [68], Theo had reported that the human child exhibits its ubiquitous and important about 24-hour rhythms with a delay after birth. His data have gained from chronomics from the analysis of time structures, a development comparable to the mapping of genes – genomics – both chronomics and genomics spawned by genetics [14]. Chronomics is a time-structurally qualified physiological genomics, based on time series analyzed for rhythms (as well as, whenever the data density will permit, for chaos, and, whenever time series length will permit, for trends). To Theo's lasting credit, he systematically distanced himself from single sample spotchecks.

Theo Hellbrügge's contributions illustrate a solidly founded now widely distributed conceptual structure resting on a productive life's work available again in his own words [9]. A few graphs and a few numbers (e.g., for rhythms with their periods and other characteristics) can meaningfully in time summarize thousands or millions of data [10,14].

With one of his colleagues [7], we can summarize how Hellbrügge's original evidence has borne many fruits in preventive health care:

• some in ethology as a method to account for the development of children,

• mother-infant-interactions as a decisive requisite of social development, the topic of the last symposium he sponsored in October 2004

• preverbal communication, as a condition for early speech promotion, especially for infants with impaired hearing,

• the plasticity of the infant's brain as a neurobiological basis for early health promotion,

• enriching integration of infant and child as part of a socially intact community,

• preventive medical-check ups aiming at an early diagnosis of abnormality,

• earliest diagnosis of risks as a condition of PREhabilitation – which he called rehabilitation, to gain a financial niche for his actions in existing laws.

Hellbrügge's conference on chronomes [14] showed advanced chronobiologic and chronomic follow-ups on what he had discovered many decades earlier [67,68]. His contributions encouraged further investigations. Furthermore a cosmic view, visualized already by Bernhard de Rudder [69], another chronobiologically active predecessor of Theo in pediatrics in Munich, is being added to child development in health and disease [14]. Preventive pediatrics can gain in Theo's footsteps a thoroughly grounded, scientific, biological yet also transdisciplinary basis. Theo's social pediatrics focuses upon the obviously handicapped child. A follow-up could focus on risks that are not obvious but may be detected chronomically as alterations of blood pressure and heart rate series. These alterations represent greater dangers than hypertension itself [65,70-72]. It is the pediatrician's opportunity to nip them in the bud in Theo's footsteps.

## References

[B1] Cornélissen G, Masalov A, Halberg F, Richardson JD, Katinas GS, Sothern RB, Watanabe Y, Syutkina EV, Wendt HW, Bakken EE, Romanov Y (2004). Multiple resonances among time structures, chronomes, around and in us. Is an about 1.3-year periodicity in solar wind built into the human cardiovascular chronome?. Human Physiology.

[B2] Halberg F, Cornélissen G, Stoynev A, Ikonomov O, Katinas G, Sampson M, Wang ZR, Wan CM, Singh RB, Otsuka K, Sothern RB, Sothern SB, Sothern MI, Syutkina EV, Masalov A, Perfetto F, Tarquini R, Maggioni C, Kumagai Y, Siegelova J, Fiser B, Homolka P, Dusek J, Uezono K, Watanabe Y, Wu JY, Sonkowsky R, Schwartzkopff O, Hellbrügge T, Spector NH, Baciu I, Hriscu M, Bakken E (2003). Season's Appreciations 2002 and 2003. Imaging in time: The transyear (longer-than-the-calendar year) and the half-year. Neuroendocrinol Lett.

[B3] Halberg F, Cornélissen G, Regal P, Otsuka K, Wang ZR, Katinas GS, Siegelova J, Homolka P, Prikryl P, Chibisov SM, Holley DC, Wendt HW, Bingham C, Palm SL, Sonkowsky RP, Sothern RB, Pales E, Mikulecky M, Tarquini R, Perfetto F, Salti R, Maggioni C, Jozsa R, Konradov AA, Kharlitskaya EV, Revilla M, Wan CM, Herold M, Syutkina EV, Masalov AV, Faraone P, Singh RB, Singh RK, Kumar A, Singh R, Sundaram S, Sarabandi T, Pantaleoni GC, Watanabe Y, Kumagai Y, Gubin D, Uezono K, Olah A, Borer K, Kanabrocki EA, Bathina S, Haus E, Hillman D, Schwartzkopff O, Bakken EE, Zeman M (2004). Chronoastrobiology: proposal, nine conferences, heliogeomagnetics, transyears, near-weeks, near-decades, phylogenetic and ontogenetic memories. Biomed Pharmacother.

[B4] Richardson JD, Paularena KI, Belcher JW, Lazarus AJ (1994). Solar wind oscillations with a 1.3-year period. Geophys Res Lett.

[B5] Mursula K, Zieger B (2000). The 1.3-year variation in solar wind speed and geomagnetic activity. Adv Space Res.

[B6] Hufeland CW (1798). Die Kunst das menschliche Leben zu verlaengern.

[B7] Schneeweiss B (2003). Prologue from a colleague. Neuroendocrinol Lett.

[B8] Hellbrügge T, v Wimpffen H (1973). Die ersten 365 Tage im Leben eines Kindes [The First 365 Days of a Child's Life].

[B9] Hellbrügge T (1994). Erlebte und bewegte Kinderheilkunde: Wissenschaftliche und praktische Grundlagen zur Gründung des Instituts und des Lehrstuhls für Soziale Pädiatrie und Jugendmedizin der Universität München.

[B10] Halberg E, Halberg Francine, Halberg J, Halberg F (1979). Forging chronobiology and pediatrics as well as geriatrics: a birthday greeting for Theodor Hellbrügge. Int J Chronobiol.

[B11] Halberg F (1989). Dem Begründer der Chronopädiatrie: Von der Sorge um das behinderte Kind zur Pädiatrie des zweiten Kindesalters: Nachtrag zum 70. Geburtstag von Theodor Hellbrügge. Der Kinderarzt.

[B12] Cornélissen G, Halberg F, Syutkina EV, Watanabe Y, Otsuka K, Maggioni C, Mello G, Perfetto F, Tarquini R, Haen E, Johnson D, Schwartzkopff O (2000). From Theodor Hellbrügge to pre-habilitation, chronopediatrics and chronomics. Int J Prenat Perinat Psychol Med.

[B13] Halberg F, Cornélissen G, Syutkina EV, Watanabe Y, Otsuka K, Maggioni C, Mello G, Perfetto F, Tarquini R, Haen E, Schwartzkopff O (2001). A chronopediatric pioneer who practices prehabilitation: a tribute to Theodor Hellbrügge on his 80th birthday. Pädiatrie und Grenzgebiete.

[B14] Cornélissen G, Schwartzkopff O, Niemeyer-Hellbrügge P, Halberg F, (Eds) (2003). Time structures – chronomes – in child development. International Interdisciplinary Conference, Nov. 29–30, 2002, Munich, Germany. Neuroendocrinol Lett.

[B15] Reindl K, Falliers C, Halberg F, Chai H, Hillman D, Nelson W (1969). Circadian acrophases in peak expiratory flow rate and urinary electrolyte excretion of asthmatic children: phase-shifting of rhythms by prednisone given in different circadian system phases. Rass Neurol Veg.

[B16] Bingham C, Arbogast B, Cornélissen Guillaume G, Lee JK, Halberg F (1982). Inferential statistical methods for estimating and comparing cosinor parameters. Chronobiologia.

[B17] Arbogast B, Lubanovic W, Halberg F, Cornélissen G, Bingham C (1983). Chronobiologic serial sections of several orders. Chronobiologia.

[B18] Arbogast B, Arbogast H, Halberg F, Hallek M, Hellbrügge T (1984). The chronobiology of the EEG and methods for analysis in health and in convulsive disorder. Abstracts from the International Workshop on Chronobiologic Technologies, Como, Sept. 27–28, 1984. Chronobiologia.

[B19] Arbogast B, Lubanovic W, Halberg F, Cornélissen G, Bingham C, Haus E, Kabat H (1984). Imputations derived from the single cosinor and the chronobiological serial section. Chronobiology 1982–1983.

[B20] Kleiser B, Halberg F, Cornélissen G, VanValkenburg C (1984). Plasma dehydröpiandrosterone (DHEA) and its timing in relation to DHEA-sulfate (DHEA-S) in schizophrenia and health. Biological Rhythms and Medications, Proc 1st Montreux Conf Chronopharmacol, Montreux, Switzerland.

[B21] Kleiser B, Halberg F, Cornélissen G, VanValkenburg C, Reinberg A, Smolensky M, Labrecque G (1984). Quantitative chronopharmacodynamic endpoint in health and schizophrenia: timing of plasma dehydroepiandrosterone (DHEA) vs. DHEA-sulfate. Annual Review of Chronopharmacology, Proc 1st Int Montreux Conf of Biological Rhythms and Medications, Montreux, Switzerland.

[B22] Arbogast H, Sothern R, Halberg F (1985). Macroscopic differentiation by plasma LH of Stein-Leventhal syndrome (S) from clinical health (H) quantified by cosinor. Chronobiologia.

[B23] Beyzavi K, März W, Sothern RB, Halberg F (1985). Circadiseptan prominence in systolic (S) & circaseptan in diastolic (D) blood pressure (BP) & heart rate (HR) of a 20-year-old woman. Chronobiologia.

[B24] Carandente F, Ferrario VF, Halberg F, März W, Cornélissen G, Schaffer EM, Ferrario G, Giani P (1985). Infradian, mostly circaseptan profiles for the diagnosis and treatment of blood pressure elevation. Abstract, 2nd Eur Mtg on Hypertension, Ric Sci Ed Perm Suppl.

[B25] Halberg F, Cornélissen G, Ahlgren A, Sothern RB, März W, Cagnoni M, Scarpelli P, Tarquini B, Halberg E Hyperbaric impact and other chronobiologic indices from self- and automatic blood pressure measurements for prevention, diagnosis and therapy. Abstract, International Symposium on Ambulatory Monitoring, Padua, Piccin.

[B26] Halberg F, Halberg E, Carandente F, Cornélissen G, März W, Halberg J, Drayer J, Weber M, Schaffer E, Scarpelli P, Tarquini B, Cagnoni M, Tuna N, Dal Palù C, Pessina AC (1986). Dynamic indices from blood pressure monitoring for prevention, diagnosis and therapy. ISAM Proc Int Symp Ambulatory Monitoring, Padua.

[B27] Halberg F, Halberg E, Cornélissen G, März W, Carandente F (1985). Automatic chronobiologic blood pressure self-monitoring in hospital, home and workplace. Ric Sci Ed Perm Suppl.

[B28] Halberg F, Halberg E, Hermida Dominguez RC, Halberg J, Cornélissen G, McCall WC, McCall VR, März W, Del Pozo Guerrero F Chronobiologic blood pressure (BP) and heart rate (HR) self-monitoring at home, workplace, school and elsewhere. IEEE/7th Ann Conf Engineering in Medicine and Biology Soc, Chicago.

[B29] Halberg F, Hermida R, Cornélissen G, Bingham C, März W, Tarquini B, Cagnoni M (1985). Toward a preventive chronocardiology. J Interdiscipl Cycle Res.

[B30] März W, Scarpelli PT, Livi R, Romano S, Cagnoni M, Cornélissen G, Halberg F (1985). Chronobiologic reference norms for time-specified measurements and circadian characteristics of systolic and diastolic blood pressure in 9-year-olds. Abstract, 2nd Eur Mtg on Hypertension, June 9-12, 1985 Ric Sci Ed Perm Suppl.

[B31] März W, Warwick WJ, Cornélissen G, Sinaiko A, Halberg F (1985). Systolic (S) & diastolic (D) blood pressure (BP) and heart rate (HR) in cystic fibrosis patients. Chronobiologia.

[B32] Scarpelli PT, März W, Cornélissen G, Romano S, Cagnoni M, Livi R, Scarpelli L, Halberg E, Halberg F Blood pressure self-measurement in schools for rhythmometric assessment of hyperbaric impact to gauge pressure "excess". Abstract, International Symposium on Ambulatory Monitoring, Padua, Piccin.

[B33] Scarpelli PT, März W, Cornélissen G, Romano S, Livi R, Scarpelli L, Halberg E, Halberg F, Dal Palù C, Pessina AC (1986). Blood pressure self-measurement in schools for rhythmometric assessment of hyperbaric impact to gauge pressure "excess". ISAM Proc Int Symp Ambulatory Monitoring, Padua.

[B34] Scarpelli PT, März W, Halberg F, Cornélissen G, Livi R, Scarpelli L, Romano S, Cagnoni M (1985). Chronobiologic tracking of circadian systolic and diastolic blood pressure mesor and hyperbaric impact for early self-evaluation and responsibility for self-help in health care. Abstract, 2nd Eur Mtg on Hypertension Ric Sci Ed Perm Suppl.

[B35] Sinaiko A, März W, Cornélissen G, Halberg F (1985). Chronobiologic monitoring of blood pressure (BP) in children in health & with kidney disease. Chronobiologia.

[B36] Arbogast H, Sothern R, Halberg F, Halberg F, Reale L, Tarquini B (1986). Cosinor assessment of differences in MESOR and acrophase of plasma luteinizing hormone (LH) in teenagers with Stein-Leventhal syndrome (S) and clinically healthy (H) girls. Proc 2nd Int Conf Medico-Social Aspects of Chronobiology, Florence.

[B37] Baranowska B, Lazicka-Frelek M, Migdalska B, Zgliczynski S, Zumoff B, Rosenfeld RS, Cornélissen G, Arbogast B, Eckert E, Halberg F, Halberg F, Reale L, Tarquini B (1986). Circadian timing of serum cortisol in patients with anorexia nervosa. Proc 2nd Int Conf Medico-Social Aspects of Chronobiology, Florence.

[B38] Halberg F, Cornélissen G, Bingham C, Tarquini B, Mainardi G, Cagnoni M, Panero C, Scarpelli P, Romano S, März W, Hellbrügge T, Shinoda M, Kawabata Y (1986). Neonatal monitoring to assess risk for hypertension. Postgrad Med.

[B39] Halberg F, Kausz E, Winter Y, Wu J, März W, Cornélissen G (1986). Circadian rhythmic response in cold pressor test. J Minn Acad Sci.

[B40] Halberg F, McCall WC, McCall VR, März W (1986). Chronobiologic blood pressure monitoring detects reactive-, amplitude- and mesor-hypertension. Chronobiologia.

[B41] Cagnoni M, Tarquini B, Halberg F, März W, Cornélissen G, Mainardi G, Panero C, Shinoda M, Scarpelli P, Romano S, Bingham C, Hellbrügge T (1987). Circadian variability of blood pressure and heart rate in newborns and cardiovascular chronorisk. Progress in Clinical and Biological Research.

[B42] Johns KL, Halberg F, Cornélissen G, März W, Halberg F, Reale L, Tarquini B (1986). Chronobiology at the American International School in Lisbon, Portugal. Proc 2nd Int Conf Medico-Social Aspects of Chronobiology, Florence.

[B43] Keenan M, März W, Halberg F (1986). Automatic 7-day monitoring of human blood pressure (BP) in health. J Minn Acad Sci.

[B44] März W, Cornélissen G, Halberg F (1986). Ultradian structure of nightly systolic blood pressure (BP) in clinical health. J Minn Acad Sci.

[B45] März W, Halberg F (1986). Time-varying, cardiovascular risk-specified 95% prediction limits for young adults in clinical health. Chronobiologia.

[B46] Meis P, März W, Halberg F (1986). Rhythmometry of conventionally acceptable or elevated blood pressure in human pregnancy. Chronobiologia.

[B47] Panero C, Mainardi G, Halberg F, Cagnoni M, März W, Cornélissen G, Tarquini B Circadian variation of blood pressure (BP) in human neonates. Proc XVII Int Cong Pediatrics, Honolulu, Hawaii.

[B48] Pangerl A, März W, Halberg F (1986). Rapid but not abrupt transmeridian adjustment of circadian acrophase (Φ) of systolic (S) blood pressure (BP). J Minn Acad Sci.

[B49] Scarpelli PT, Romano S, Cagnoni M, Livi R, Scarpelli L, Croppi E, Bigioli F, März W, Halberg F, Halberg F, Reale L, Tarquini B (1986). Blood pressure self-measurement as part of instruction in the Regione Toscana. Proc 2nd Int Conf Medico-Social Aspects of Chronobiology, Florence.

[B50] Tarquini B, Lombardi P, Pernice LM, Andreoli F, März W, Cornélissen G, Halberg F (1986). Ultradian structure of gastric pH at night. J Minn Acad Sci.

[B51] Wendt H, März W, Cornélissen G, Halberg F (1986). Circadian & ultradian blood pressure (BP) rhythmometry also reveals nocturnal episodic elevation of BP but not of heart rate (HR). J Minn Acad Sci.

[B52] Cagnoni M, Tarquini B, Halberg F, Mainardi G, Panero C, März W, Cornélissen G, Shinoda M, Kawabata Y, Bingham C (1987). Neonatal monitoring of blood pressure and heart rate and early cardiovascular risk assessment. Biochim Clin.

[B53] Cagnoni M, Tarquini B, Halberg F, März W, Cornélissen G, Mainardi G, Panero C, Shinoda M, Scarpelli P, Romano S, Bingham C, Hellbrügge T (1987). Circadian variability of blood pressure and heart rate in newborns and cardiovascular chronorisk. Progress in Clinical and Biological Research.

[B54] Halberg F, Warwick W, Cornélissen G, März W, Wilson D, Ferencz C (1987). Chronobiologic assessment of heart rate & blood pressure in cystic fibrosis & incidence of tachycardia. Chronobiologia.

[B55] März W, Halberg F (1987). Circadian systolic and diastolic differences (CSDD) and circadian modulation of 1.7-h ultradians. Chronobiologia.

[B56] Wegmann R, Wegmann A, Wegmann-Goddijn M-A, März W, Halberg F (1987). Hyperbaric indices (HBI) assess the extent and timing of deviant blood pressure in patients under treatment. Chronobiologia.

[B57] Marques N, Marques MD, Marques R, Marques L, März W, Halberg F Circannual blood pressure variation in 4 family members: delayed adjustment after a transequatorial flight. Proc XX Int Conf Chronobiol, Tel Aviv, Israel.

[B58] Marques N, Marques MD, Marques RD, Marques LD, März W, Halberg F (1995). Delayed adjustment after transequatorial flight of circannual blood pressure variation in 4 family members. Il Policlinico, Sez Medica.

[B59] Halberg F, Cornélissen G, Katinas G, Syutkina EV, Sothern RB, Zaslavskaya R, Halberg F, Watanabe Y, Schwartzkopff O, Otsuka K, Tarquini R, Perfetto P, Siegelova J (2003). Transdisciplinary unifying implications of circadian findings in the 1950s. J Circadian Rhythms.

[B60] Hale GE (1924). Sun-spots as magnets and the periodic reversal of their polarity. Nature.

[B61] Halberg F, Cornélissen G, Otsuka K, Schwartzkopff O, Halberg J, Bakken EE (2001). Chronomics. Biomedicine and Pharmacotherapy.

[B62] Cornélissen G, Halberg F, Fiser B, Johnson P, Mitsutake G, Gigolashvili M, Chibisov SM, Katinas GS, Siegelova J, Dusek J, Otsuka K, Schwartzkopff O Geographic differences in presence/prominence of transyearly cycles in the incidence of sudden cardiac death. Biomedicine & Pharmacothearpy.

[B63] Hellbrügge T Letter to Prof. MUDr. Jarmila Siegelova.

[B64] Cornélissen G, Halberg F, Schwartzkopff O, Delmore P, Katinas G, Hunter D, Tarquini B, Tarquini R, Perfetto F, Watanabe Y, Otsuka K (1999). Chronomes, time structures, for chronobioengineering for "a full life". Biomed Instrum Technol.

[B65] Otsuka K, Cornélissen G, Schwartzkopff O, Bakken EE, Halberg F, Burioka N, Katinas GS, Kane R, Regal PJ, Schaffer E, Sonkowsky R, Patterson R, Engebretson M, Brockway B, Wang ZR, Delmore P, Halpin C, Sarkozy S, Wall D, Halberg J (2003). Clinical chronobiology and chronome-geriatrics: At variance with recommendations of subsequent guidelines, yet focusing indeed on pre-hypertension in the physiological range. Biomed Pharmacother.

[B66] Jacobson J, Jacobson C (2004). Evaluation of hearing loss in infants and young children. Pediatric Annals.

[B67] Hellbrügge T (1960). The development of circadian rhythms in infants. Cold Spr Harb Symp Quant Biol.

[B68] Hellbrügge T, Lange JE, Rutenfranz J, Stehr K (1964). Circadian periodicity of physiological functions in different stages of infancy and childhood. Ann NY Acad Sci.

[B69] De Rudder B (1952). Grundriss einer Meteorobiologie des Menschen: Wetter- und Jahreszeiteneinflüsse Dritte neubearbeitete Auflage Mit 56 Abbildungen.

[B70] Müller-Bohn T, Cornélissen G, Halhuber M, Schwartzkopff O, Halberg F (2002). CHAT und Schlaganfall. Deutsche Apotheker Zeitung.

[B71] Halberg F, Cornélissen G, Schwartzkopff O, Hardeland R, Ulmer W (2003). Messung und chronobiologische Auswertung der Variabilitäten von Blutdruck und Herzfrequenz zur Prophylaxe schwerwiegender Krankheiten. Proc Leibniz Soz.

[B72] Halberg F, Cornélissen G, Schack B (2004). Self-experimentation chronomics for health surveillance and science, also transdisciplinary civic duty?. Behavioral and Brain Sciences.

